# Evaluation of two-step iterative resampling procedure for internal validation of genome-wide association studies

**DOI:** 10.1186/1471-2105-15-S10-P34

**Published:** 2014-09-29

**Authors:** Guolian Kang, Wei Liu, Cheng Cheng, Carmen L Wilson, Geoffrey Neale, Jun J Yang, Kirsten K Ness, Leslie L Robison, Melissa M Hudson, Kumar Srivastava

**Affiliations:** 1 Department of Biostatistics, St. Jude Children’s Research Hospital, Memphis, TN 38105, USA; 2 Department of Epidemiology and Cancer Control, St. Jude Children’s Research Hospital, Memphis, TN 38105, USA; 3 Hartwell Center for Bioinformatics and Biotechnology, St. Jude Children’s Research Hospital, Memphis, TN 38105, USA; 4 Department of Pharmaceutical Sciences, St. Jude Children’s Research Hospital, Memphis, TN 38105, USA

## Background

Genome-wide association (GWA) studies have successfully identified many common genetic variants associated with complex diseases over the past decade. The standard method for validating the top single nucleotide polymorphisms (SNPs) identified in GWA studies is to identify and replicate the findings in external cohorts. However, in cases of rare diseases like retinoblastoma and Ewing sarcoma that have prevalence rates of about 0.000001, it can be difficult to find an external validation cohort within a reasonable time frame. Even when disease outcomes are not rare, it can be hard to find a suitable external validation cohort.

## Materials and methods

In these situations, resampling approaches or two-stage validation approaches such as the one proposed by Yang et al. [1] have been used to identify SNPs associated with the outcome of interest. The two-stage validation approach proposed in [1] is based on dividing the original cohort equally into discovery and replication cohorts. A SNP is considered discovered and replicated if the p-values corresponding to the tests are significant at levels α_1_ and α_2_ in the two stages. This process is repeated 100 times and a SNP is considered to be “validated” if the SNP is discovered and replicated at least 10 times in 100 repetitions. However, there was no justification provided for the 50:50 split and the statistical properties of the approach were also not evaluated. In this paper, we undertook extensive simulation studies to assess the effect of various split ratios, various type I error cut-offs for stage I, and different cut-offs used in 100 repetitions on the performance of the approach regarding the overall type I error and statistical power. Two independent simulation studies, one for the binary phenotype and the other for continuous phenotype, were conducted.

## Results

Our simulation results indicate that, for GWA studies, the two-stage approach with a 70:30 split and a cut-off of 20 in 100 replications is reasonable as it is able to maintain an overall type I error at *α*_1_*α*_2_ and provides near “optimal” power for internally validated SNPs. We then applied this approach to the two GWA study examples and validated the SNPs identified in the original GWA studies. The first GWA study was conducted to identify SNPs associated with obesity (evaluated as a binary phenotype) in survivors of childhood cancer treated with cranial radiation, while the aim of the second study was to identify SNPs associated with heart failure (evaluated as a continuous phenotype) in cancer survivors who received cardiotoxic therapy.
